# Hypotensive Effect of Nanomicellar Formulation of Melatonin and Agomelatine in a Rat Model: Significance for Glaucoma Therapy

**DOI:** 10.3390/diagnostics10030138

**Published:** 2020-03-03

**Authors:** Massimo Dal Monte, Maurizio Cammalleri, Salvatore Pezzino, Roberta Corsaro, Nicola Pescosolido, Paola Bagnoli, Dario Rusciano

**Affiliations:** 1 Department of Biology, University of Pisa, via San Zeno, 31, 56127 Pisa, Italy; maurizio.cammalleri@unipi.it (M.C.); paola.bagnoli@unipi.it (P.B.); 2 Sooft Research Center, Viale Andrea Doria, 21, 95125 Catania, Italy; salvatore.pezzino@sooft.it (S.P.); corsaro.roberta@gmail.com (R.C.); dario.rusciano@sooft.it (D.R.); 3 Department of Clinical Internal, Anesthesiological and Cardiovascular Sciences, Sapienza University of Rome, Piazzale Aldo Moro, 5, 00185 Rome, Italy; pescosol@tiscali.it

**Keywords:** melatoninergic agents, tonometry, normotensive and hypertensive eyes, animal model

## Abstract

Background: Melatoninergic agents are known to reduce intraocular pressure (IOP). The present study was performed to evaluate the effect of nanomicellar formulations of melatoninergic agents on IOP in the rat. Methods: Tonometry was used to measure IOP in eyes instilled with melatonin or agomelatine. Ocular hypertension was induced by the injection of methylcellulose in the anterior chamber. Results: Melatonin formulated in nanomicelles had a longer lasting hypotonizing effect on IOP with respect to melatonin in saline. Nanomicellar formulations of melatonin and agomelatine, either alone or in combination, had lowering effects that did not depend on their concentration or their combination, which, however, resulted in an increased duration of the hypotonizing effect. The duration of the lowering effect was further increased by the addition of lipoic acid. Conclusions: We demonstrated the effective hypotonizing activity of melatonin and agomelatine in combination with lipoic acid. Although results in animals cannot be directly translated to humans, the possibility of developing novel therapeutical approaches for patients suffering from hypertensive glaucoma should be considered.

## 1. Introduction

Physiological levels of intraocular pressure (IOP), ranging from 9 to 21 mmHg in humans, basically depend on the balance between the rate of aqueous humor production by the epithelium of the ciliary body and the rate of its main drainage through the trabecular meshwork and the canal of Schlemm into the vein circulation; when IOP is higher than normal, this is referred to as ocular hypertension [1].

Glaucoma is a blinding disease representing the most common neurodegenerative pathology as it is expected to affect about 111 million people worldwide by 2040 [2]. Although the pathogenesis of glaucoma is not fully understood, the increase in IOP is a major risk factor for the disease, ultimately leading to compression and degeneration of the optic nerve [3]. The main approved treatments for glaucoma rely on the use of drugs that are able to reduce IOP, including prostaglandin analogs, β-adrenergic blockers, α-adrenergic agonists, carbonic anhydrase inhibitors, Rho kinase inhibitors, and cholinergic agonists, either as monotherapy or combination therapy [4]. These drugs reduce IOP by about 20% to 40%, which is usually efficacious in preventing (or at least delaying) pressure-induced vision loss [5]. However, these therapies are not without adverse effects and their efficacy is also subject to a reduction after prolonged use. In addition, there are patients who continue to experience glaucoma progression despite IOP reduction, thus indicating the necessity of additional therapeutic approaches [6]. For instance, neuroprotective molecules, eventually preventing retinal ganglion cell degeneration, have been reported to halt or delay glaucoma progression when used alone or in combination with IOP-lowering drugs [7]. If the same molecule could act as an IOP-lowering drug and neuroprotectant at the same time, then it would be of great help for patient compliance. For instance, the α2A selective agonist brimonidine not only reduces IOP in glaucoma patients [8], but also exerts neuroprotective effects in models of retinal degeneration [9], although its scarce tolerability can limit its use [10].

Melatonin is a neurohormone released during the dark period of the day not only by the pineal gland, but also by ocular structures including the retina, lens, iris, and ciliary body. In the eye, melatonin serves several functions; it exerts antiapoptotic and antioxidant activities and, in particular, decreases IOP, thus suggesting that melatonin or its analogues could be used to treat glaucoma [1]. In particular, topical application of saline formulations of melatonin or melatonin analogues such as methoxycarbonylamino-*N*-acetyltryptamine or agomelatine (commonly used as anti-depressant) [11] significantly reduces IOP [12,13,14]. Both melatonin and agomelatine have higher efficacy in reducing IOP in hypertensive than in normotensive eyes, suggesting that melatonin and its analogues can have a more effective hypotensive activity when hypertensive glaucoma is established [12,14]. In humans, oral melatonin is effective in reducing basal IOP in subjects with normotensive IOP and in patients undergoing cataract surgery [15,16], while oral agomelatine has been proven to reduce IOP in glaucoma patients [17]. Melatonin has also been demonstrated to exert neuroprotective effects in *in vivo* and *in vitro* models of retinal ganglion cell death induced by glutamate excitotoxicity [18], suggesting that melatoninergic agents can be used to reduce both ocular hypertension and degenerative processes in glaucomatous eyes. It is worth noting that melatonin is safe and well tolerated, even at high doses, and no adverse effects have been described indicating that melatonin could be an attractive pharmacological candidate to treat glaucoma [19]. However, the potential of melatonin-based therapies needs to be better evaluated. In addition, information about possible synergistic effects of melatonin with its analogues is still not available. The additional fact that melatonin acts as a potent free radical scavenger and antioxidant is indicative of the possibility that oxidative stress and reactive oxygen species can contribute to its degradation, thus limiting its half-life [20]. In this respect, the activity of melatonin can be potentiated by the addition of antioxidants among which lipoic acid is a fatty acid acting as a natural antioxidant that has been previously used to increase the antioxidant activity of melatonin [21]. Whether lipoic acid can influence the effect of melatonin or its analogues on IOP in glaucoma models has not been investigated so far. In this respect, lipoic acid, *per se*, does not influence IOP as demonstrated in a mouse model of spontaneously developed hypertensive glaucoma [22].

Here, we evaluated whether single drops containing melatonin or agomelatine either alone or in combination could affect the basal IOP in normotensive eyes of the rat. In the first step, we compared the efficacy of melatonin formulated in saline with that formulated in nanomicelles. Then, nanomicellar formulations of melatonin were compared with those of agomelatine either alone or in combination without or with lipoic acid. Finally, in hypertensive eyes of a rat model of increased IOP, we compared the hypotensive effects of the mixture of melatonin and agomelatine without or with lipoic acid. 

## 2. Materials and Methods

### 2.1. Animals and Experimental Model of Hypertensive Eyes

**Figure 1:**
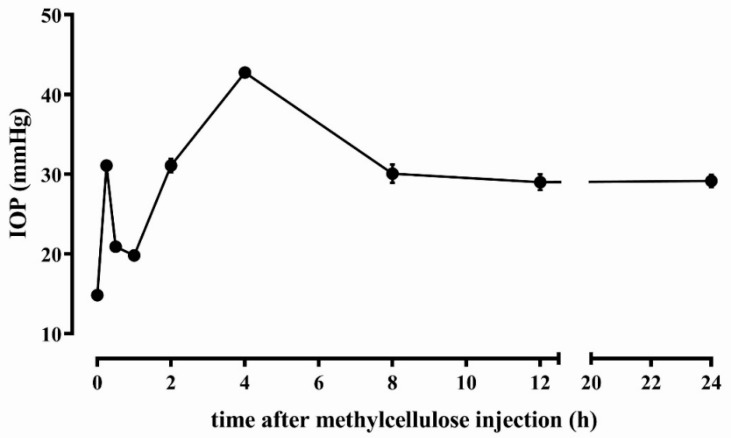
Time course of intraocular pressure progression in rats injected with 15 µL of 2% methylcellulose in the anterior chamber (n = 9 rats, 18 eyes).

Animals were used in compliance with the Association for Research in Vision and Ophthalmology statement for the Use of Animals in Ophthalmic and Vision Research. The present study also adheres to the European Communities Council Directive (2010/63/UE) and the Italian guidelines for animal care (DL 26/14). The experimental protocol was approved by the Commission for Animal Wellbeing of the University of Pisa (protocol n. 133/2019-PR, February 14, 2019). According to 3Rs principles for ethical use of animals in scientific research, all efforts were made to reduce both the number of animals and their suffering. Rats (Sprague Dawley strain) were obtained from Charles River Laboratories Italy (Calco, Italy) and a breeding colony was established in the animal facility of the Department of Biology. Rats were maintained under standard laboratory conditions of 12 h cycles of light and dark, with free access to food and water. All rats were acclimatized to handling and tonometry for 1 week. Thirty-six rats, either male or female (8 weeks old), were used and of these, 27 were used to evaluate the effect of different concentrations and formulations on the normotensive IOP, while 9 rats were used as a model of hypertensive IOP. For each rat, both eyes were treated with either saline or eye drops. IOP was measured in both eyes by tonometry. The model of increased IOP was obtained by injecting the anterior chamber of the rat eye with methylcellulose, a molecule that leads to ocular hypertension by mechanically blocking the outflow of the aqueous humor [23]. The injection of viscoelastic substances causes a viscosity-dependent ocular hypertension by blocking the trabecular meshwork channel, thus rendering the methylcellulose model similar to the microbead injection models [24]. Rats were anesthetized with an intraperitoneal injection of pentobarbital (30 mg/kg) and injected in both eyes with 15 µL of 2% methylcellulose in sterile saline through an 18G needle under a microscope. As shown in Figure 1, after the injection, we observed an early, transient elevation in IOP, due to the increase in the volume of the aqueous humor after the injection, with a return to the basal level within 1 h. A second peak was reached within 4 h after methylcellulose administration as a consequence of the trabecular tissue clotting, after which a steady IOP was maintained until 24 h. Twenty-four hours after methylcellulose injection, the rats were utilized for the evaluation of hypotensive effects of the melatonin and agomelatine mixture without or with lipoic acid.

### 2.2. Preparation of Eye Drops

The formulation of melatonin in saline was prepared by dissolving melatonin (Molekula, Darlington, UK) at 0.4% in sterile isosmotic phosphate buffered saline. The formulation of melatonin and agomelatine (Molekula) in nanomicelles (Soluplus^®^, 115.000 g/mol; BASF, Ludwigshafen, Germany) was prepared by the direct dissolution method. Nanomicelles were diluted at 11.5% *w*/*v* in Tris buffer at pH 7.4. Melatonin and agomelatine were then added, keeping the system under magnetic stirring at room temperature for 24 h to allow complete and homogeneous dispersion. The formulation was sterilized by filtration through 0.22 μm sterile membranes (Minisart; Sartorius, Gottinga, Germany). Osmolarity was measured by an osmometer (Osmomat 3000; Gonotec, Berlin, Germany) and corrected to 300 mOsm by the addition of NaCl. The mean particle size and polydispersity index were determined using a NanoSizer ZS90 (Malvern Panalytical, Malvern, UK). Samples were diluted ten-fold with water before analysis. No differences were observed between loaded and unloaded micelles in terms of mean size. Blank nanomicelles had an average size of 61.78 ± 1.61 nm and a polydispersity index of 0.068 ± 0.022; the micelles in the three formulations showed almost identical values. The stability of these micelles was monitored at 1, 3, and 6 months, and their characteristics remained unchanged. The nanomicelles were transparent, slightly opalescent as compared with water. Lipoic acid (T5625, Sigma-Aldrich, St. Louis, MO, USA) was dissolved at 0.15% in the 0.4% melatonin and agomelatine nanomicellar formulation.

### 2.3. Administration of Eye Drops and Measurement of Intraocular Pressure

Rats were divided in different experimental groups (3 rats/experimental group), each receiving saline, melatonin at 0.4% in saline, or additional formulations in nanomicelles (melatonin at 0.4%, melatonin at 0.8%, agomelatine at 0.4%, agomelatine at 0.8%, melatonin and agomelatine both at 0.4%, melatonin and agomelatine both at 0.8%, and melatonin and agomelatine both at 0.4% with lipoic acid at 0.15%). Each rat received a single drop (10 µL) in both eyes. Before and at different times after eye drop instillation, IOP was measured by tonometry using an Icare TonoLab instrument (Icare Finland Oy, Helsinki, Finland). For each eye, IOP was determined as an average of 10 measurements. No evidence of corneal or conjunctival toxicity, such as signs of chemical trauma, iatrogenic corneal toxicity, inflammation or conjunctivitis, were observed after eye drop applications. This is in agreement with a previous study demonstrating that eye drops based on melatoninergic agents did not induce toxicity in a battery of standard ocular surface irritation studies [25].

### 2.4. Statistical Analysis

Statistical analysis was performed using Prism 8.02 (GraphPad Software, Inc., San Diego, CA, USA). Statistical significance was evaluated using either two-way analysis of variance (ANOVA) followed by Bonferroni’s multiple comparison post-test or unpaired Student’s *t* test, as appropriate. The results are expressed as mean ± standard error of the mean (SEM) of the indicated n values. Differences with *p* < 0.05 were considered significant.

## 3. Results

### 3.1. Normotensive IOP: Effect of Melatonin in Saline vs. Melatonin in Nanomicellar Formulation

**Figure 2:**
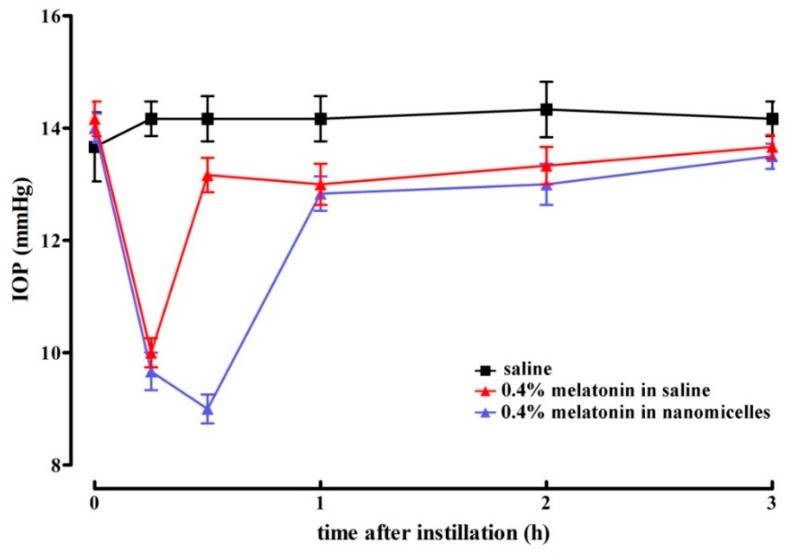
Lowering effect of melatonin formulated at 0.4% in saline or in nanomicelles in rats with normotensive intraocular pressure. No difference in the magnitude of lowering effect could be assessed among the different formulations. The nanomicellar formulation increased the duration of the hypotonizing effect. Values represent the mean ± standard error of the mean (SEM) of data from 3 rats (6 eyes).

In line with previous results [12,14], Figure 2 shows that melatonin at 0.4% formulated in saline reduced IOP in normotensive eyes. As also shown in Figure 2, nanomicellar formulations of melatonin at 0.4% showed a significantly longer lasting hypotonizing effect with respect to saline formulation (30 min *vs.* 15 min), although in the presence of comparable lowering effects. The statistical analysis of data shown in Figure 2 is detailed in Table 1.

### 3.2. Normotensive IOP: Effect of Melatonin and Agomelatine Formulated in Nanomicelles either Alone or in Combination, without or with Lipoic Acid

**Figure 3:**
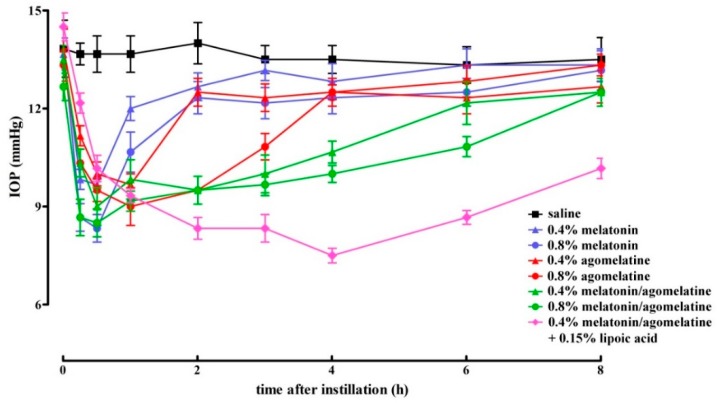
Lowering effect of different formulations of melatonin and agomelatine either alone or in combination without or with lipoic acid in rats with normotensive intraocular pressure. No difference in the magnitude of lowering effect could be assessed among the different concentrations and formulations. An increase in the concentration of melatonin and agomelatine, their combination or the addition of lipoic acid increased the duration of the lowering effect. Values represent the mean ± SEM of data from 3 rats (6 eyes).

In normotensive eyes, we evaluated whether the hypotonizing effect of melatonin and its analogue agomelatine, instilled either alone or in combination, could be potentiated by increasing their concentration or by the addition of lipoic acid. Figure 3 shows the hypotonizing effect on IOP, while Table 2 details the statistical analysis of data shown in Figure 3. The time peak of the lowering effect on IOP was reached within 30 min after instillation and did not depend on either concentrations or formulations of the compounds. Increasing concentrations of either melatonin or agomelatine were found to significantly prolong the duration of their hypotensive effect (30 min with melatonin at 0.4% *vs.* 1 h with melatonin at 0.8% and 1 h with agomelatine at 0.4% *vs.* 3 h with agomelatine at 0.8%). The lowering effect of melatonin in combination with agomelatine was significantly more prolonged with respect to each compound alone at both 0.4% (almost 4 h *vs.* 30 min with melatonin at 0.4% or 1 h with agomelatine at 0.4%) and 0.8% (almost 6 h *vs.* 1 h with melatonin at 0.8% or 3 h with agomelatine at 0.8%). Lipoic acid addition to melatonin and agomelatine at 0.4% significantly increased the duration of the hypotonizing effect of the mixture (from 4 to 8 h).

### 3.3. Hypertensive IOP: Effect of Nanomicellar Formulations with Combined Melatonin and Agomelatine, without or with Lipoic Acid

**Figure 4:**
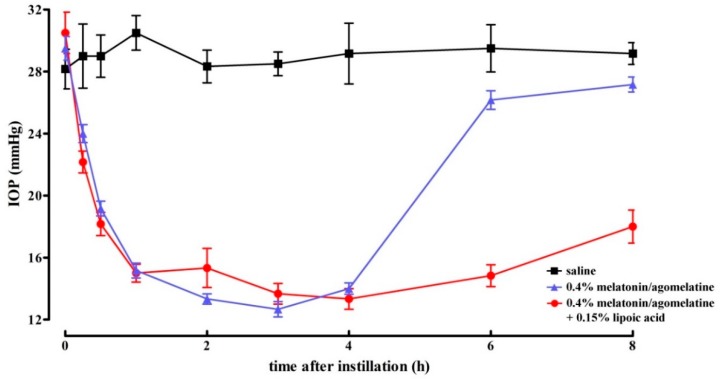
Lowering effect of melatonin and agomelatine at 0.4% without or with lipoic acid formulated in nanomicelles in rats with hypertensive intraocular pressure. No difference in the magnitude of lowering effect could be assessed after the addition of lipoic acid that, however, increased the duration of the mixture efficacy. Data represent the mean ± SEM of data from 3 rats (6 eyes).

In a rat model of increased IOP after methylcellulose injection in the anterior chamber, we found that the mixture of melatonin and agomelatine at 0.4%, without or with lipoic acid drastically decreased IOP (to levels that were not different from normotensive values) within 1 h after instillation. Figure 4 shows the hypotonizing effect on IOP, while Table 3 details the statistical analysis of data shown in Figure 4. As shown in Figure 4, the magnitude of the effect of the mixture did not depend on the presence of lipoic acid, whereas the effect duration was almost doubled by its addition (4 h without lipoic acid *vs.* 8 h with lipoic acid). By comparing the data in Table 3 with those in Table 2, it can be noticed that the lowering effect of melatonin and agomelatine was higher in rats with hypertensive IOP than in rats with normotensive IOP (−55.5% and −32.1%, respectively, *p* < 0.01).

## 4. Discussion

Glaucoma represents a group of diseases leading to loss of vision as a consequence of optic nerve degeneration. In glaucoma, an increase in IOP represents one of the main risk factors and hypotonizing therapies are the main approved treatments for glaucoma patients. However, the approved therapies can present adverse effects in many patients and show a loss of efficacy after prolonged use. In addition, reducing IOP does not always improve the disease since additional pathogenetic mechanisms, such as neurodegenerative processes, are involved in determining glaucoma and its progression. In this respect, glaucoma patients can take advantage of molecules exerting IOP-lowering effects and neuroprotective actions at the same time [26]. If one considers that several patients continue to experience glaucoma progression despite IOP reduction, then novel therapies need to be urgently developed [6] and basic research is actively involved in investigating molecules with potential application as anti-glaucoma agents.

Here, we confirm previous findings demonstrating that melatoninergic agents exert hypotonizing effects on IOP [12,13,14,15,16,17]. The present findings also show that, when melatonin is formulated in nanomicelles, the duration of its effect is drastically increased, suggesting that melatoninergic agents administered in nanotechnology formulations can have longer lasting hypotonizing effects with respect to saline formulations whose efficacy is limited over time [12,14]. This would occur by enhancing compound bioavailability to eye tissues as the result of the mucoadhesiveness of the nanomicellar preparation, its larger cargo capacity, and the controlled drug release [27]. Although no nanomicellar formulations of melatonin have been used so far to increase melatonin delivery to the eye, there is evidence that formulations with liposomes, which represent an additional drug delivery system, increase the duration of the hypotonizing effect of the melatonin analogue methoxycarbonylamino-*N*-acetyltryptamine in the rabbit eye [28].

The possible role of melatonin was suggested about 25 years ago after the observation that melatonin controls the aqueous humor dynamics [29]. Then, the hypotonizing effect of melatoninergic agents was demonstrated in *in vivo* models of elevated IOP. In fact, topical application of melatoninergic agents dissolved in saline has been demonstrated to reduce normotensive IOP with an effect ranging from 10% to 50% reduction [12,13,14,25], which is in line with the present findings. In particular, agomelatine-based eye drops are as effective in reducing normotensive IOP as additional molecules commonly used in the treatment of glaucoma patients including the prostaglandin F2a analog latanoprost and the α2A-adrenergic receptor agonist brimonidine, thus indicating that melatoninergic agents could expand the current repertoire of drugs used to counteract ocular hypertension [14].

Melatoninergic agents used in combination with IOP-lowering drugs increases their efficacy in reducing IOP. For instance, both melatonin and its analogue methoxycarbonylamino-*N*-acetyltryptamine in combination with either the non-selective β-adrenergic receptor antagonist timolol or with brimonidine are able to potentiate the hypotensive effect of the adrenoceptor ligands [13]. However, no information on possible potentiation of the hypotonizing efficacy by combining melatoninergic agents is available so far. As shown here, melatonin and agomelatine, when topically administered in combination, display a longer lasting effect than when administered alone, suggesting the possibility that their combined action could overcome the need of repeated daily instillations. On the one hand, this could be due to optimized receptor stimulation as agomelatine has higher affinity for melatonin receptors than melatonin itself [25,30]. On the other hand, melatonin receptors form homo- or heterodimers that are differentially stimulated by melatonin or its analogues [1], thus generating less or more pronounced hypotonizing effects.

As also shown here, the hypotonizing effect of melatonin and agomelatine is higher in hypertensive than in normotensive eyes. In fact, melatonin reduces normotensive IOP by approximately 20% and hypertensive IOP by approximately 33%, in line with previous findings in the rabbit model [12,14]. This is presumably due to the different compartmentalization of melatonin, its receptors, or its synthetic enzymes between hypertensive and normotensive eyes [1].

The present study also demonstrates that the addition of lipoic acid increases the duration of the lowering effect of melatonin and agomelatine by approximately 50%, suggesting that the presence of antioxidants can increase the half-life of melatoninergic agents. The mechanisms through which melatoninergic agents reduce IOP are not fully understood and could involve several mechanisms in addition to the activation of melatonin receptors [1]. Among these mechanisms, the potent antioxidant activity of melatonin likely plays a role in reducing IOP. In this respect, melatonin could act by stimulating antioxidant enzymes or inhibiting prooxidant enzymes, but also by directly scavenging free radicals that, in turn, irreversibly convert melatonin to its metabolites [20]. Lipoic acid acts as a broad-spectrum antioxidant molecule and is devoid of lowering effect on IOP [22,31]. It acts presumably by sparing melatoninergic agents from oxidation, thus slowing down the reduction in their concentration similarly to what is found in aged rats, in which lipoic acid prevents the oxidation of reduced glutathione, one of the main antioxidant molecules naturally occurring within the cell [32].

Although limited literature describes the hypotonizing effect of melatoninergic agents in humans, there is evidence that oral administration of melatonin or its analogues reduces IOP in subjects with normotensive IOP, as well as in patients with glaucoma [15,16,17]. As proof-of-concept that eye drops combining melatonin and agomelatine can be effective in patients suffering from hypertensive glaucoma, an anecdotic observation from a patient affected by bilateral primary open-angle glaucoma under maximum tolerated medical therapy demonstrates that oral melatonin (2 mg given three times per day) or topical galenic formulations consisting of melatonin 1% in association with agomelatine 1% (three times per day for one week) drastically reduce IOP by more than 50% in both eyes [33]. This observation supports the data from basic research and suggests the possibility that melatonin is effective in reducing IOP when topically administered in combination with agomelatine. However, the efficacy of melatoninergic compounds in patiens with glaucoma needs to be fully evaluated in an appropriate experimental study involving human subjects suffering from hypertensive glaucoma.

## 5. Conclusions

In conclusion, significant preclinical gaps need to be bridged before developing therapies that can be used in humans to effectively block glaucoma progression. In this respect, preclinical studies could represent an invaluable step to ensure safety and efficacy of possible therapeutic interventions, although the difficulty in translating the results from animal models to humans. The present finding that nanomicellar formulations of melatonin and agomelatine added with lipoic acid are effective in reducing IOP in hypertensive eyes represents the proof-of-concept that combinations of melatoninergic agents formulated in carriers are able to enhance their bioavailability and added with antioxidants could become useful as a treatment (or as an adjuvant in treatments with standard therapies) for patients suffering from hypertensive glaucoma.

## Figures and Tables

**Table 1:** Statistical analysis of data shown in Figure 2. Differences were calculated with respect to the intraocular pressure measured in rats instilled with saline.

Time	0.4% Melatonin in Saline	0.4% Melatonin in Nanomicelles
0	3.7%	2.4%
15 min	−29.4% ^1^	−31.8% ^1^
30 min	−7.1%	−36.4% ^1,2^
1 h	−8.3%	−9.5%
2 h	−6.9%	−9.3%
3 h	−3.5%	−4.7%
4 h	−3.5%	−7.1%
6 h	3.7%	2.4%
8 h	3.7%	1.3%

^1^*p* < 0.001 *vs.* saline and ^2^ *p* < 0.001 *vs.* 0.4% melatonin in saline. Statistical analysis was performed using two-way ANOVA followed by Bonferroni’s multiple comparison post-test.

**Table 2:** Statistical analysis of data shown in Figure 3. Differences were calculated with respect to the intraocular pressure measured in rats instilled with saline.

Time	0.4% Melatonin	0.4% Agomelatine	0.4% Melatonin/Agomelatine	0.8% Melatonin	0.8% Agomelatine	0.8% Melatonin/Agomelatine	0.4% Melatonin/Agomelatine + 0.15% Lipoic Acid
0	−1.2%	0	−2.4%	0	−3.6%	−8.4%	4.8%
15 min	−28.9% ^1^	−19.2% ^2^	−25.3% ^1^	−37.3% ^1^	−25.3% ^1^	−37.3% ^1^	−11.0% ^2^
30 min	−30.1% ^1^	−27.7% ^1^	−34.9% ^1^	−39.1% ^1^	−30.5% ^1^	−37.8% ^1^	−25.6% ^1^
1 h	−12.2%	−29.3% ^1^	−28.1% ^1,3^	−21.9% ^4,5^	−34.2% ^1^	−32.9% ^1^	−31.7% ^1^
2 h	−9.5%	−10.7%	−32.1% ^1,6,7^	−11.9%	−32.1% ^1,8^	−32.1% ^1,9^	−41.0% ^1^
3 h	−2.4%	−8.7%	−25.9% ^1,6,7^	−9.9%	−19.8% ^10^	−28.4% ^1,11^	−38.3% ^1^
4 h	−5.0%	−7.4%	−21.0% ^5,8,11^	−8.7%	−7.4%	−25.9% ^1,10,11^	−44.4% ^1,11,12^
6 h	0	−7.5%	−8.7%	−6.2%	−3.6%	−18.9% ^2,10,11,13^	−27.5% ^1,14,15^
8 h	−1.3%	−6.1%	−7.4%	−2.4%	−1.3%	−7.4%	−24.7% ^1,14,16^

^1^*p* < 0.001 *vs.* saline; ^2^ *p* < 0.05 *vs.* saline; ^3^ *p* < 0.01 *vs.* 0.4% melatonin; ^4^ *p* < 0.01 *vs.* saline; ^5^ *p* < 0.05 *vs.* 0.4% melatonin; ^6^ *p* < 0.001 *vs.* 0.4% melatonin; ^7^ *p* < 0.01 *vs.* 0.4% agomelatine; ^8^ *p* < 0.05 *vs.* 0.4% agomelatine; ^9^ *p* < 0.01 *vs.* 0.8% melatonin; ^10^ *p* < 0.05 *vs.* 0.8% melatonin; ^11^ *p* < 0.05 *vs.* 0.8% melatonin; ^12^ *p* < 0.001 *vs.* 0.8% melatonin; ^13^ *p* < 0.05 *vs.* 0.4% melatonin/agomelatine; ^14^ *p* < 0.001 *vs.* 0.4% melatonin/agomelatine; ^15^ *p* < 0.05 *vs.* 0.8% melatonin/agomelatine; ^16^ *p* < 0.01 *vs.* 0.8% melatonin/agomelatine. Statistical analysis was performed using two-way ANOVA followed by Bonferroni’s multiple comparison post-test.

**Table 3:** Statistical analysis of data shown in Figure 4. Differences were calculated with respect to the intraocular pressure measured in rats instilled with saline.

Time	0.4% Melatonin/Agomelatine	0.4% Melatonin/Agomelatine + 0.15% Lipoic Acid
0	4.7%	8.3%
15 min	−17.2% ^1^	−23.6% ^2^
30 min	−33.9% ^2^	−37.3% ^2^
1 h	−50.3% ^2^	−50.8% ^3^
2 h	−52.9% ^2^	−45.9% ^2^
3 h	−55.5% ^2^	−52.0% ^2^
4 h	−52.0% ^2^	−54.3% ^2^
6 h	−26.0%	−49.7% ^2,3^
8 h	−6.9%	−38.3% ^2,3^

^1^*p* = 0.05 *vs.* basal; ^2^ *p* < 0.001 *vs.* basal; ^3^ *p* < 0.001 *vs.* 0.4% melatonin and agomelatine. Statistical analysis was performed using two-way ANOVA followed by Bonferroni’s multiple comparison post-test.
